# Understanding the Beat-to-Beat Variations of P-Waves Morphologies in AF Patients During Sinus Rhythm: A Scoping Review of the Atrial Simulation Studies

**DOI:** 10.3389/fphys.2019.00742

**Published:** 2019-06-18

**Authors:** Dimitrios Filos, Dimitrios Tachmatzidis, Nicos Maglaveras, Vassilios Vassilikos, Ioanna Chouvarda

**Affiliations:** ^1^Lab of Computing, Medical Informatics and Biomedical Imaging Technologies, School of Medicine, Aristotle University of Thessaloniki, Thessaloniki, Greece; ^2^3rd Cardiology Department, Aristotle University of Thessaloniki, Thessaloniki, Greece; ^3^Department of Industrial Engineering and Management Sciences, Northwestern University, Evanston, IL, United States

**Keywords:** atrial fibrillation, multiple P-wave morphologies, sinus rhythm, computational models, simulation, scoping review

## Abstract

The remarkable advances in high-performance computing and the resulting increase of the computational power have the potential to leverage computational cardiology toward improving our understanding of the pathophysiological mechanisms of arrhythmias, such as Atrial Fibrillation (AF). In AF, a complex interaction between various triggers and the atrial substrate is considered to be the leading cause of AF initiation and perpetuation. In electrocardiography (ECG), P-wave is supposed to reflect atrial depolarization. It has been found that even during sinus rhythm (SR), multiple P-wave morphologies are present in AF patients with a history of AF, suggesting a higher dispersion of the conduction route in this population. In this scoping review, we focused on the mechanisms which modify the electrical substrate of the atria in AF patients, while investigating the existence of computational models that simulate the propagation of the electrical signal through different routes. The adopted review methodology is based on a structured analytical framework which includes the extraction of the keywords based on an initial limited bibliographic search, the extensive literature search and finally the identification of relevant articles based on the reference list of the studies. The leading mechanisms identified were classified according to their scale, spanning from mechanisms in the cell, tissue or organ level, and the produced outputs. The computational modeling approaches for each of the factors that influence the initiation and the perpetuation of AF are presented here to provide a clear overview of the existing literature. Several levels of categorization were adopted while the studies which aim to translate their findings to ECG phenotyping are highlighted. The results denote the availability of multiple models, which are appropriate under specific conditions. However, the consideration of complex scenarios taking into account multiple spatiotemporal scales, personalization of electrophysiological and anatomical models and the reproducibility in terms of ECG phenotyping has only partially been tackled so far.

## Introduction

Over a century ago, Atrial Fibrillation (AF) had been recognized as the most common arrhythmia in adults (Nishida and Nattel, 2014). It is characterized by chaotic atrial activation leading to impaired atrial myocardial function. It is easily recognized on a surface electrocardiogram by lack of atrial depolarization represented by P-wave, quivering isoelectric line and irregular ventricular activation represented by QRS-complexes, which lead to contractile dysfunction (Pellman and Sheikh, 2015). In the case that AF converts to normal sinus rhythm (SR) within 7 days, it is classified as “paroxysmal,” while in the case of it lasting more than 7 days or more than a year it is classified as “persistent” or “long-standing persistent,” respectively (Camm et al., 2012a; Kirchhof et al., 2016).

Atrial fibrillation is associated with increased morbidity and mortality as it is widely recognized as a risk factor for embolic stroke and heart failure (HF) exacerbation (Camm et al., 2012b). On the other hand, numerous risk factors have been correlated with the development of AF. Aging doubles AF risk per decade, whereas gender influences the incidence of AF with males having 1.5 fold risk to develop AF (Andrade et al., 2014). Most cases of persistent and permanent AF are related to hypertensive, valvular, ischaemic or other types of structural heart disease, while lone AF represents only 15% of the cases (Markides and Schilling, 2003).

Initiation and maintenance of the abnormal rhythm in both lone AF and AF secondary to structural heart disease are supposed to require pathophysiological remodeling of the atria (Pellman and Sheikh, 2015). Remodeling can be grouped into three categories that include:

(i) electrical remodeling, including modulation of L-type Ca2+ current, various K+ currents, and gap junction function,
(ii) structural remodeling, including changes in tissues properties, size, and ultrastructure, and
(iii) autonomic remodeling, including altered sympathovagal activity, and hyperinnervation (Pellman and Sheikh, 2015).

Electrical, structural, and autonomic remodeling all contribute to creating an AF-prone substrate which can produce AF-associated electrical phenomena including a rapidly firing focus, complex multiple reentrant circuits, or rotors (Nattel, 2002). Since the 1960s, the most popular theory has held that AF consists of multiple wavelets of functional re-entry (Moe et al., 1964). Conditions that increase the atrial size or decrease the wavelength (by decreasing the conduction velocity or refractory period or both) permit multiple wavelets and promote AF (Rensma et al., 1988).

Multiple factors have been found to relate and contribute to AF initiation or perpetuation mechanisms. Ionic currents and gap junction function are considered to be fundamental parts of electrical remodeling occurring in atrial cardiomyocytes, which leads to reduced action potential (AP) duration and refractoriness and affects conduction velocity and wavelength, both known determinants of AF initiation and perpetuation (Gaspo et al., 1997; Pellman and Sheikh, 2015). Atrial enlargement has been associated with AF many decades ago (Henry et al., 1976). Moreover, scarring, fibrosis, and increased atrial size observed in HF patients, are related to conduction delay and refractory period prolongation (Sanders et al., 2003). The autonomic nervous system (ANS) exerts significant control over cardiac electrophysiology, while it has been proposed that patterns of baseline autonomic nerve activity are essential in the development of pacing-induced sustained AF (Shen et al., 2011). There is a suggestion that the increased sympathetic activity leads to heterogeneous changes in atrial refractoriness, which in turn favors reentrant waves that generally contribute to the maintenance of AF (Olgin et al., 1998). On the other hand, ablation of the ganglionated plexi can also improve long-term AF symptoms (Katritsis et al., 2013). Additionally, the muscular sleeves of the pulmonary veins (PVs) have been identified as a source of tachyarrhythmias and atrial premature beats that could trigger paroxysms of AF (Haïssaguerre et al., 1998). These findings lead to a unifying theory that focal tachycardias (mostly originating from the PVs) promote atrial remodeling and they are required to trigger and maintain a substrate capable of multiple wavelet reentry (Veenhuyzen et al., 2004). AF causes electrophysiological changes in the atrial myocardium itself, which might explain the progressive nature of the arrhythmia. In a landmark study, the perpetuation of AF was accompanied by a shortening of the atrial refractory period inducing electrophysiologic changes that promote further AF. These include electrical, contractile and structural modifications to the atria that have collectively become known as atrial remodeling and the authors concluded that “AF begets AF” (Wijffels et al., 1995). However, most of the findings are based on electrophysiological studies making the reproducibility of the experiments a challenging case.

The interplay of different factors contributing to AF and the inherent complexity of the biological systems, necessitates the development of computational models across different levels, from the cell, tissue, and patient level. Computational modeling can help study and shed light on the mechanisms of AF, and thus new therapeutic approaches can be developed and tested before applying them to patients (Jacquemet, 2016). The main advantage of computational modeling is that researchers have access to all levels of interest and thus they can perform experiments in controlled and repeatable conditions, which is a limitation of experimental and clinical research. The increased evidence on the importance and the effectiveness of computational modeling in several fields of science, including cardiac electrophysiology, in association with the advances in the computational speed (Virag et al., 2012) and the availability of such infrastructure, were the primary catalysts on the increased interest in the field of computational modeling during the recent years. Overall, computational modeling can link the phenomenological organ level findings, such as findings in the electrocardiography (ECG), and the actual mechanisms at smaller scales.

As regards the AF patients, the analysis of P-wave during SR is of clinical value, since P-wave morphology is affected by the site of origin of the SR beat and the atrial conduction routes (Platonov, 2012). The analysis of P-waves can reveal information related to the prediction of AF initiation (Martínez et al., 2014a) or the success of the pulmonary vein isolation (Huo et al., 2015). Also, the analysis of the P-wave morphology variability, on a beat-to-beat basis, also revealed differences between healthy subjects and AF patients (Censi et al., 2016; Filos et al., 2017). However, the direct correlation of those ECG findings with the underlying substrate modifications is not well understood. The motivation behind this research was to find what mechanisms may explain the difference in the percentage of the primary/secondary P-wave morphologies observed in ECG signals of paroxysmal AF vs. normal subjects, and more importantly whether these mechanisms were studied, quantified and reproduced via simulation studies. To serve this need, we designed a scoping review, (a) to map the existing literature in the field of atrial models and their multifaceted properties/components related to AF, and (b) to synthesize this knowledge toward explaining the link between observable AF-related P-wave morphologies and computational atrial models of AF. Finally, this work attempts to identify the research gaps and to make recommendations for future research.

## Methodology

We conducted a scoping review adopting the methodology proposed by Arksey and O’Malley (2005) and Peters et al. (2015). The aim of scoping reviews is to map existing literature in a particular field as well as to synthesize the knowledge. The definition proposed by Colquhoun et al. (2014) states that “A scoping review or scoping study is a form of knowledge synthesis that addresses an exploratory research question aimed at mapping key concepts, types of evidence, and gaps in research related to a defined area or field by systematically searching, selecting, and synthesizing existing knowledge.”

### Objective

We focused on the identification of the mechanisms, either on cell, tissue or organ level, which can describe the variation of the P-wave morphologies in AF patients during SR while we tried to investigate the existence of computational models which can reproduce those mechanisms. The following research questions were posed:

(1) How was AF modeled?
(2) Which computational models were proposed for the description of patients prone to AF during SR?
(3) How was the initiation of AF episodes modeled?
(4) Were those models able to reproduce the multiple P-wave morphologies that appear in AF patients during SR?

The primary outcomes that are of interest in this scoping review are the characteristics of the AF substrate which describe atrial remodeling occurring as a result of AF. The comprehension of such mechanisms may lead to get more insights on the prediction of the AF onset and thus facilitate the management of patients in terms of the prevention and adaptation of the therapeutic approach.

### Inclusion Criteria

We applied the PICO (Population, Intervention, Comparator, and Outcome) eligibility criteria. Regarding the population, we considered all AF studies without distinction between human and animal models. All types of AF were considered, including Asymptomatic, Paroxysmal, Persistent, or Permanent. As for the intervention, any level of computational models that enabled the simulation of the atrial activation was eligible for inclusion. Models focusing on the cell or tissue level, as well as on the organ level were examined.

Studies comparing simulated and real ECG phenotyping or comparison of different computational models were included in the literature review as well as studies without a comparator, such as theoretical models. Finally, any publication, such as articles, conference proceedings, editorials and chapters in textbooks, published after 1998 until 1 of March 2018, written in English was included in the review, whereas all review articles were excluded.

### Search Strategy

A two steps search strategy was adopted to map the existing research on this topic. Two databases, which are extensively used in biomedical sciences, were searched (PubMed and Scopus). Initially, a limited search in both databases was undertaken followed by an analysis of the text words contained in the title and the abstract as well as on the indexed terms of the article. The second search step included the extensive literature search based on the keywords extracted in the first step and the manual inspection of the studies.

**FIGURE 1:**
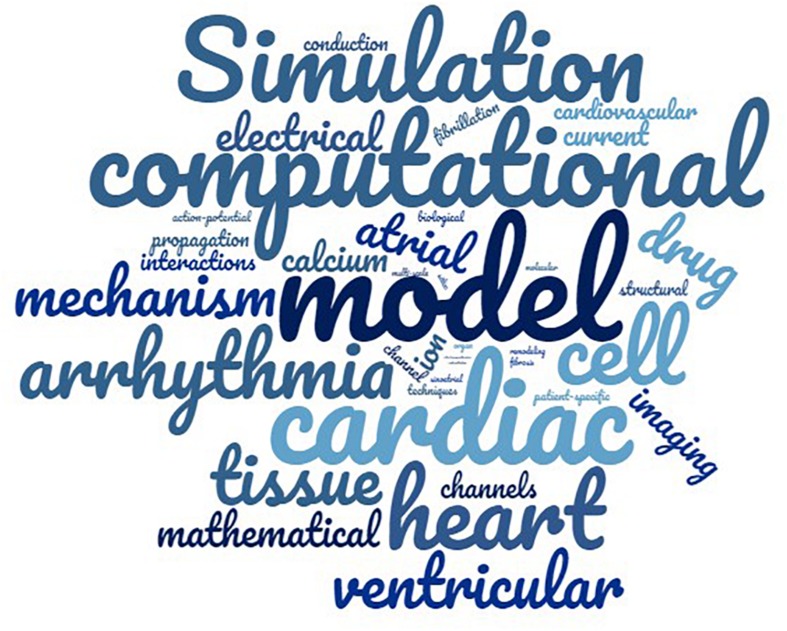
Word cloud of the most frequent words, where the highest the font the higher the frequency. For the purposes of the visualization the terms model, models, modeling, and modeling were grouped under the term “model.” As depicted, the most used words are model, cardiac, computational, simulation, heart, and with frequencies 16.6, 11.16, 7.2, 6.5, and 5.7%, respectively.

**FIGURE 2:**
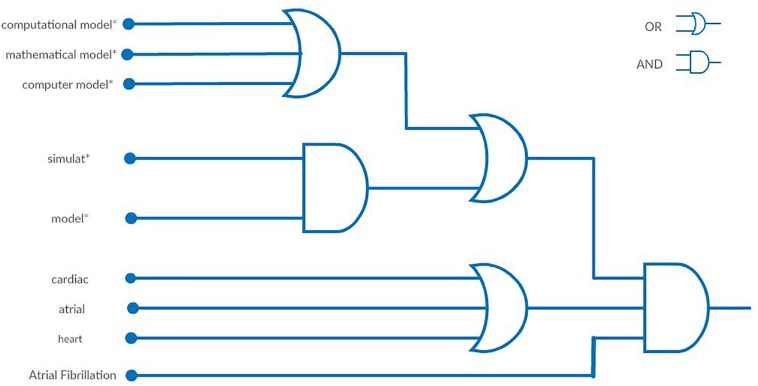
Visual representation of the final query using logic gates. The final query is described as: {[(“computational model^*^”[Title/Abstract] OR “mathematical model^*^”[Title/Abstract] OR “computer model^*^”[Title/Abstract]) OR (simulat^*^[Title/Abstract] AND model^*^[Title/Abstract])] AND (“cardiac”[Title/Abstract] OR “atrial”[Title/Abstract] OR “heart”[Title/Abstract])} AND “atrial fibrillation”[Text Word].

Regarding the text analysis made during the first step, review articles, published during the last 10 years, in which the words “cardiac” and “simulation” were included in their title or abstract were selected, as they are considered to cover the field of the current review. As a result, 115 and 163 articles were found in PubMed and Scopus, respectively. After removing the duplicates, 216 review papers were screened, whereas only 99 review articles were found to be relevant to our study. Subsequently, a text analysis of the titles and the abstract was conducted. A table of the most frequent words and phrases was produced, while equivalent words were identified (e.g., modeling and modeling). The word cloud depicted in Figure 1 presents in a visual way the most frequent words. The most common phrases were found to be “computation model^*^,” “mathematical model^*^,” or “computer model^*^,” which describe the same process. On the other hand, the word “model^*^” and “simulat^*^” appeared in the same abstract, but not necessarily accompanied, to describe the same approach as the phrases above. The asterisk (^*^) following a word describes all of the words starting with the phrase before it [e.g., model(s), modeling are described as model^*^]. Based on these findings we formulated the first two blocks of Figure 2, which are connected to an OR logical gate. The terms “cardiac,” “atrial,” and “heart” were extracted as the most frequent words, that are related to the description of the model or the tissue of interest. Finally, the term “Atrial Fibrillation” was not extracted by the text analysis but it was used since each article was focused on AF.

These findings led to the creation of the search query that was used in the second step of the literature review (Figure 2). In this step, the words and phrases identified were searched in the abstract, the title or the indexed keywords. The whole literature search, its evaluation, and categorization were conducted independently by two reviewers (DF and DT). After the completion of the evaluation, in case of disagreement, the two reviewers worked together to reach a consensus.

## Results

**FIGURE 3:**
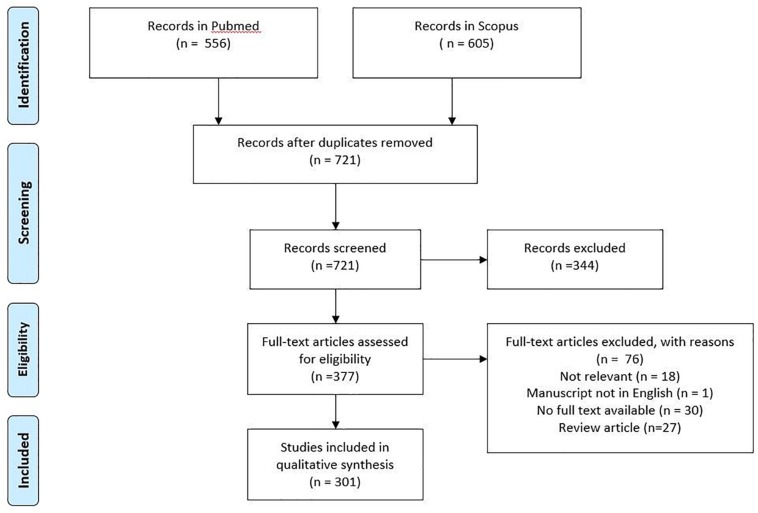
PRISMA flowchart of the study selection procedure.

**FIGURE 4:**
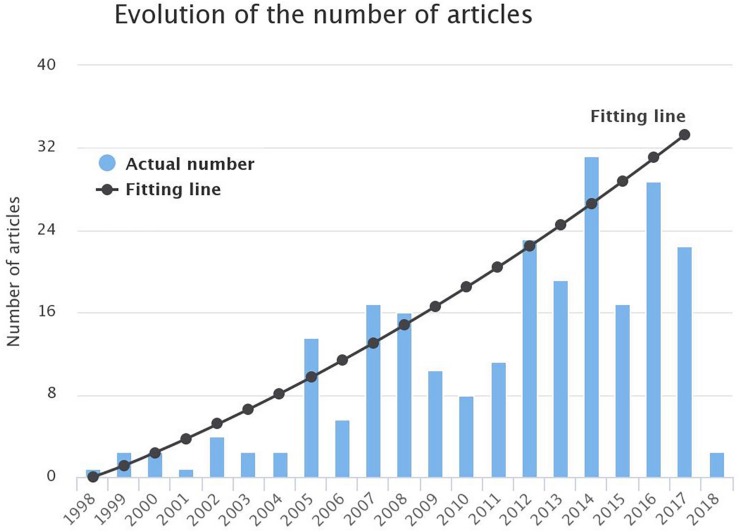
Evolution of the number of publications in the field of AF modeling from 1998 till 1st March 2018. A parabolic line was found to fit better the data depicted in the bars ($R^{2}=0.7609$).

A total number of 721 articles (excluding duplicates) was raised. Titles and abstracts were screened to check whether they are relevant to the research question or not. Approximately half of them (377) were eligible for full-text review. Seventy-six articles were excluded, resulting in a total of 301 articles which were finally included in the review (Figure 3). The observation of Figure 4 reveals a continuous increase in the number of articles published over time.

### Modeling Components

The interplay of multiple factors can lead to the initiation and perpetuation of the arrhythmia (Kirchhof et al., 2016). In this section, the existing literature in the field of atrial models is presented while a categorization of the articles was made based on the factors that are addressed in each simulation study.

#### Model Experimental Context

##### Animal studies

**FIGURE 5:**
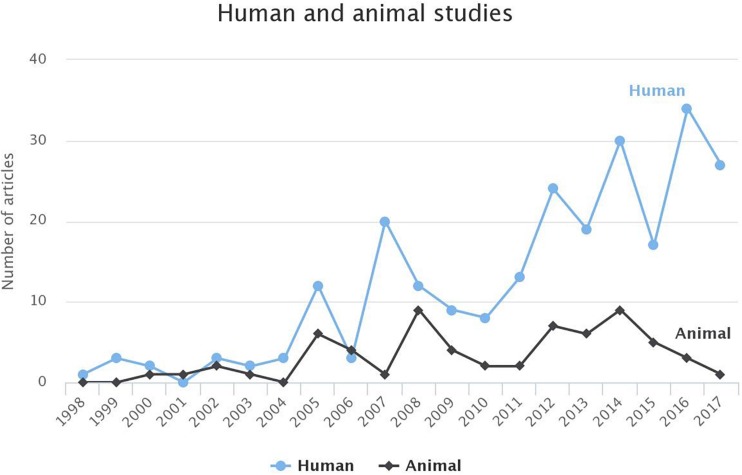
Comparison of the studies based on human or animal models for AF for each year. For 2018, until 1st of March 2018, the studies targeting humans and animals were 3 and 0, respectively. To avoid any false perception those data have not been visualized.

While in the first years, the ratio between the studies focusing on humans or animals was approximately 1:1, during the last decade, an increased interest in human studies is observed (Figure 5). The animal studies can improve our understanding of the pathophysiological properties of AF while their findings can be useful in human studies.

A detailed model of the canine atrial cell is presented by Ramirez et al. (2000) which is applicable in both normal and electrically remodeled cellular substrate. The following years, additional animal models were proposed able to describe the sheep (Goodman et al., 2005; Butters et al., 2013), rat (Majumder et al., 2016) or other animals’ atrial myocardium (Aslanidi et al., 2009b). A novel computational model of the canine left atrium (LA) and PVs is proposed by Cherry et al. (2007) and Aslanidi et al. (2011a) through which an attempt was made to understand the arrhythmogenicity of PV and how re-entries and their geometry can promote their excitation. The differences between the right atrium (RA) and LA in terms of the electrophysiological properties in the animals are described in Aslanidi et al. (2009a) and in Xia et al. (2010).

Aslanidi et al. (2008) studied the effects of Ca^2+^ concentration in the cell of the RA in terms of AF initiation. According to the simulation performed in a cell and tissue level, no crucial effect of intracellular Ca^2+^ concentration on reentries has been observed as its results are reversible. Furthermore, Kneller et al. (2005) studied the influence of antiarrhythmic drugs in the canine atrium, and found that the inhibition of pure Na current during AF can terminate the arrhythmia.

Other mechanisms were also addressed in animal models before being incorporated in the human ones, such as the effect of ANS (Kneller et al., 2002) and of the antiarrhythmic drugs (Comtois et al., 2008; Almquist et al., 2010; Aguilar-Shardonofsky et al., 2012; Colman et al., 2014; Aguilar et al., 2015; Varela et al., 2016).

Vigmond et al. (2001) presented a morphologically realistic atrial model where all the major anatomical structures of the atrium, including fiber orientation, muscle structures such as crista terminalis and pectinate muscles and the orifices of the veins and valves, were considered and scaled to a canine atrium level. The resulting model did not consist of an exact representation of the atria. However, it reflected the interrelations between atrial anatomical structures. Zhao et al. (2012) proposed a detailed structural model of the sheep atria which was based on *ex vivo* analysis of the atrial chambers. The details of the fiber orientations and myofiber architecture were incorporated into an anatomical model. The simulation performed in normal and electrically remodeled conditions, confirm the unique roles of crista terminalis, pectinate muscles and Bachman’s bundle on the activation time as well as the differences on the electrical propagation through the posterior LA.

In addition to animal models, many studies are taking into account findings from experiments performed on animals, to modify the human model. The goal of those studies extends from studying the effects of drug therapy (Gomez et al., 2005; Ehrlich et al., 2008) to impact of gene mutations (Hancox et al., 2014; Syeda et al., 2016).

##### Evaluation of model output

Computational modeling is by nature an approximation of reality and several approaches have been adopted to evaluate their output. However, most of the studies employed *in silico* experiments, rather than a direct evaluation of the simulation outputs with real recordings. On the other hand, some of the articles included a comparison between the *in silico* and the *in vivo* findings, based on electrophysiological findings (Courtemanche et al., 1998, 1999; Ramirez et al., 2000; Wettwer et al., 2004; Kneller et al., 2005; Kuijpers et al., 2007; Comtois et al., 2008; Lombardo et al., 2016). Furthermore, the reproducibility of the simulated signals, electrograms or ECG, with the real recordings is discussed in the context of solving the forward (Ogawa et al., 2007; Burdumy et al., 2012) or inverse problem (Pedron-Torrecilla et al., 2016). Finally, the need for personalized anatomical and electrical models is highlighted toward the highest reproducibility of the results (Krueger et al., 2013c; Roney et al., 2016).

#### Model Electrophysiological Components at Different Scales

##### Electrophysiological cell models in humans

The core of each study is the electrophysiological model which describes the cellular ionic properties and transmembrane currents. A plethora of different approaches has been proposed to simulate electrical activity in human atria.

Initially, a common approach was the use of models describing the ventricular activity, such as those of Beeler and Reuter (1977) or Luo and Rudy (1991), to simulate atrial excitation (Virag et al., 2002). On the other hand, membrane kinetics describing the atrial myocardium in animals, such as in canines, have been applied in the human atrium (Ruchat et al., 2007b, 2007c), hypothesizing similar ionic properties.

**FIGURE 6:**
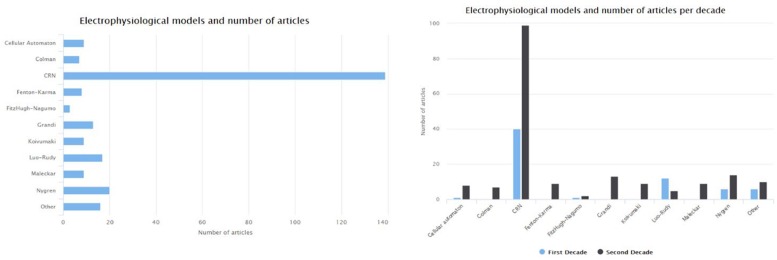
Different electrophysiological models and the number of studies which use them.

The CRN (Courtemanche et al., 1998) and Nygren et al. (1998) models are the first attempts to describe the ionic mechanism of the atrial myocytes in humans. The CRN model was based on adaptations made on a model describing ventricular myocytes. The Maleckar et al. (2009) model refined the description of Nygren model’s K${}^{2+}$ current, while the Koivumäki et al. (2011) extended the Nygren model in order to improve the description of the Ca^2+^ dynamics. The Grandi et al. (2011) model focuses on patients with chronic AF (cAF) and in SR. Figure 6 depicts the number of articles focusing on the human atria that where based on each of the most frequently used electrophysiological models. The most frequently used model is the one proposed by Courtemanche et al. (1998). Compared to the first decade (1998–2007), the number of articles for AF which adopt ventricular models [such as that of Luo and Rudy (1991)] decreased to half in the second decade (2008–2018). On the other side, the newly proposed models by Koivumäki et al. (2011), Grandi et al. (2011), and Colman et al. (2016) seem to gain the research community’s acceptance.

Cherry and Evans (2008), as well as Krueger et al. (2013a) focused on the comparison of electrophysiological models. In particular, Krueger et al. (2013a) performed a comparison between CRN, Nygren, and Maleckar electrophysiological models adjusted to describe 13 different atrial regions heterogeneities, including electrical remodeling. A detailed and personalized MRI-based anatomical model, including fiber orientation detail and thorax model, was estimated for each of the eight patients to reproduce P and Ta-wave accurately. According to the results, the CRN model simulates better action potential duration (APD) in each of the atrial regions compared with the remaining two models. Furthermore, the simulated P-waves and Ta-waves morphology calculated using the CRN models fitted well with the experimental recordings in both physiological and AF-remodeled conditions. Wilhelms et al. (2013) compared the five most frequently used electrophysiological models of human atria and presented the main strengths and weaknesses of each of the models. Lombardo et al. (2016) performed a comparison between two electrophysiological models, a detailed and a simpler one. The results showed that both models reproduce the clinical recordings and the spiral waves dynamics were similar and thus, in the case that computationa cost is crucial, simple models could be used to model arrhythmias in spatially extended domains.

On the other hand, some articles (Ciaccio et al., 2017; Lin et al., 2017) adopted the use of a cellular automaton model (CA) like the one proposed by Moe et al. (1964), which was the first CA model for AF. In general, CA models describe the depolarization and repolarization of a cell based on rules of its present state as well as on its neighbors while more detailed CA models have been proposed recently (Manani et al., 2016). The main property of a CA model is that it is suitable for observation of macroscopic properties of the electrical propagation since it is a simplified model of cardiac electrophysiology and the required computational load is low.

##### Genes and mutations

Several studies reveal the occurrence of AF on a family basis which implies a genetic cause of this arrhythmia. Genetic mutations have been found to lead to modification of ionic channel functionality. The first study identified is the one of Seemann et al. (2004) where a 2D model of the RA was used to investigate the effect of a mutation in the KCNQ1 gene on the initiation of AF. In the following years, the effect of additional gene mutations or gene expressions on the initiation of AF was studied (Table 1). In all these studies, the parameters of the atrial models have been modified accordingly, to reproduce the findings from the electrophysiological studies. Most of the studies addressed the effect on the slow delayed outward rectifier potassium channel ($I_{\text{Ks}}$) (Seemann et al., 2004; Ehrlich et al., 2005; Kharche et al., 2012a) as well as on other potassium (Seemann et al., 2009) or sodium channels (Ziyadeh-Isleem et al., 2014). While most articles focus on the effects of gene expression on a cell or 2D tissue model, Whittaker et al. (2017) studied the stability of the re-entrant waves using a realistic atrial model. Finally, Ni et al. (2017a) examined six observed mutations of KCNA5 regarding their role in the electromechanical function of the atrium. Half of those mutations lead to gain-of-function resulting in a worsening of the contractile function of the atrium while the rest lead to loss-of-function of that mediated the positive inotropic effects.

**TABLE 1:** Genes identified and modeled.

**Gene**	**Articles**
KCNQ1	Seemann et al., 2004; Hong et al., 2005; Sampson et al., 2008; Kharche et al., 2012a; Mann et al., 2012; Ki et al., 2014; Hancox et al.,2014
KCNE1	Ehrlich et al., 2005; Sampson et al., 2008; Mann et al.,2012
KCNH2	Carrillo et al., 2008; Seemann et al., 2009; Mann et al.,2012
KCNJ2	Kharche et al., 2008; Aslanidi et al., 2012a; Mann et al., 2012; Whittaker et al.,2017
KCNA5	Mann et al., 2012; Ni et al.,2014, 2017a
SCN5A	Ziyadeh-Isleem et al.,2014
ANK2	Wolf et al.,2013
PITX2	Syeda et al.,2016
hERG	Loewe et al., 2014; Lutz et al.,2014
KCND3, KCNIP2, KCNH2, KCNE3, KCNE4, KCNE5, KCNJ4, and KCNJ14	Mann et al.,2012

##### Modeling drug effect on atrial cells and AF

Several types of antiarrhythmic drugs have been analyzed *in silico* regarding their performance on the termination of AF (Ehrlich et al., 2008; Sanchez et al., 2017), under SR and AF (Aslanidi et al., 2012a). Table 2 summarizes the studies focusing on each anti-arrhythmic drug category (class I to v).

**TABLE 2:** Studies focusing on each anti-arrhythmic drug category.

**Article**	**Drug category**	**Details**
Namba et al., 1999; Comtois et al., 2008; Colman et al., 2014; Aguilar et al., 2015; Syeda et al.,2016	Class I (Ia, Ib, Ic)	Na channel blockers
Marshall et al., 2012; Colman et al., 2014; Kharche et al.,2014b	class II	β-blockers
Wettwer et al., 2004; Syed et al., 2005; Ehrlich et al., 2008; Tsujimae et al., 2008; Almquist et al., 2010; Law et al., 2010; Aslanidi et al., 2012a; Duarte et al., 2013; Colman et al., 2014; Tobón et al., 2014a, 2017; Aguilar et al., 2015; Cacciani and Zaniboni, 2015; Majumder et al., 2016; Syeda et al., 2016; Varela et al., 2016; Ni et al.,2017b, 2016	class III	K channel blockers
Colman et al., 2014; Cacciani and Zaniboni,2015	class IV	Ca blockers
Krummen et al.,2012	class V	adenosine

The first study identified in this context, Namba et al. (1999) investigated the effects of a specific sodium channel blocker drug on the stability versus meandering of spiral waves in a canine atrial model.

One of the major challenges of antiarrhythmic drug therapy is the minimization of side effects, like QT prolongation and ventricular pro-arrhythmic conditions (Aguilar-Shardonofsky et al., 2012; Aguilar et al., 2015). A key concept to address these challenges is the design of drugs with atrial selectivity. Since ultrarapid delayed rectifying potassium current ($I_{\text{Kur}}$) is present in atrial and absent from ventricular myocardium, it is a justified target for AF antiarrhythmic therapy (Almquist et al., 2010). However, the effect of such a ($I_{\text{Kur}}$) blocker may be pro-arrhythmic during sinus rhythm and anti-arrhythmic during AF (Law et al., 2010). Moreover, the effect of selective (fast and slow onset) blockers of $I_{\text{Kur}}$ depends on the level of electrical remodeling (Wettwer et al., 2004; Tsujimae et al., 2008). Although $I_{\text{Kur}}$ is diminished in cAF, the $I_{\text{Kur}}$ blockers show prominent significance in cAF treatment, as investigated *in silico* in a number of theoretical drugs in both SR and cAF conditions (Ellinwood et al., 2017a, 2017b).

Along this line, Ni et al. (2017b) studied *in silico* the effects of the multi-channel blockers on atrial and ventricular activation, and found that the synergistic use of a specific and potentially selective $I_{\text{Kur}}$ potassium channel blocker (acacetin) and a $I_{N\,A}$ blocker seems to have no significant impact on QT prolongation or on ventricular activation. As suggested, the synergetic antiarrhythmic treatment leads to more effective termination of atrial reentries (Aguilar et al., 2015; Ni et al., 2016), while ventricular side effects in the ventricles are limited. Furthermore, the relationship between drug concentration and AF termination is also considered in other studies (Duarte et al., 2013; Tobón et al., 2014a, 2017).

Colman et al. (2014) and Varela et al. (2016) studied the effectiveness of the antiarrhythmic therapy on termination of the wave breaks near the PVs. The latter study discusses the effect of the multi-channel class III antiarrhythmic drugs in combination with atrial heterogeneities on the termination of AF, using detailed 3D anatomical models of human atria. Additionally, the role of the antiarrhythmic drugs in the prevention of AF initiation due to bradycardia (Cacciani and Zaniboni, 2015) or how isoproterenol can induce AF (Krummen et al., 2012) were also studied.

A fundamental approach to AF treatment is β-blockers usage to control ventricular response. The chronic use of such treatment can lead to drug-induced remodeling. Marshall et al. (2012) studied these pharmacological remodeling effects of β-blockers on specific potassium currents in the human atrium. Kharche et al. (2014b) discussed this pharmaceutical remodeling and observed that chronic use of β-blockers suppresses AF by APD and effective refractory period (ERP) prolongation, with potential anti-arrhythmic consequences.

##### Autonomic nervous system and the role of ganglia

The effect of vagal tone on atrial arrhythmogenesis has been reported in several studies (Vigmond et al., 2004). Modifying the ion channel conductance, Ashihara et al. (2002) simulated the two branches of the ANS and used the Luo-Rudy-1 model to examine why vagally mediated AF lasts more than sympathetic mediated AF. Interestingly, it was found that sympathetic tone promotes spiral waves and restrains their breakup. In contrast, vagal tone promotes spiral wave breakup. A significant limitation of this study is that the modifications on the ion channels were speculative as there was insufficient information from clinical data.

A significant number of studies incorporates, in the electrophysiological models, the effect of Acetylcholine to model the vagal activation and study its effect on AF (Comtois and Nattel, 2011; Voigt et al., 2013). Acetylcholine (Ach) is a neurotransmitter used in vagal action studying, while its concentration affects the APD (Vigmond et al., 2004). Experimental findings reveal increased vagal activity during hemodialysis (HD) sessions suggesting that enhanced acetylcholine concentration can lead to ERP shortening and depolarization prolongation (Vincenti et al., 2014). Kneller et al. (2002) used the RNC canine atrial model, and the Ach effect was incorporated based on a novel description of the Ach-induced current ($I_{\text{K,ACh}}$) that is included in all the studies which consider the vagal influence on AF. Hwang et al. (2016) considered three different approaches for the distribution of the ACh concentration on the atrium: (1) random distribution, (2) ACh concentration within the four ganglionated plexi (GP) areas, and (3) using the octopus hypothesis where eight nerves with gradient ACh concentration originate from the GPs and spread through the atrium. The octopus hypothesis was adopted in order to examine the effect of both branches of ANS (sympathetic and parasympathetic nervous system) (Hwang et al., 2017). The parasympathetic system was modeled as an increase of the $I_{\text{KACh}}$ in the ganglionated plexi and their nerves in the LA 3D model while the sympathetic system was modeled as an increase of the L-type calcium current. ANS stimulation can induce triggering activity and PV automaticity, leading to local re-entries in the LA-PV junction.

#### AF Remodeling

Atrial fibrillation is a progressive disease, where at the first stages the episodes are short and infrequent (paroxysmal AF), and later on, they are more frequent and more prolonged (persistent or permanent AF). As AF progresses to more stable types, different parameters of the electrophysiological models are modified to reflect the AF-induced electrical remodeling. These changes on ion currents and conductance are based on findings from experimental studies (Courtemanche et al., 1999; Zhang et al., 2005). The majority of the 301 studies included in this review focused on more stable types of AF while only about 10% of them directly referred to Paroxysmal AF (Ashihara et al., 2002; Gong et al., 2007; Severi et al., 2010; Duarte et al., 2013; Calvo et al., 2014; Vincenti et al., 2014; Voigt et al., 2014; Berenfeld, 2016).

The electrically remodeled substrate favors the initiation and the perpetuation of AF. APD shortening and decreased conduction velocity lead to reduced wavelength. However, the mechanisms for AF initiation and perpetuation may differ (Chang and Trayanova, 2016) which is the subject of several studies (Krogh-Madsen et al., 2012; Colman et al., 2013; Koivumäki et al., 2014; Lee et al., 2016). Furthermore, studies included the extent of the electrical remodeling in each atrium (Luca et al., 2015), concerning its influence on the duration of AF episodes. Among the articles included in this review, 44 articles were found to study AF initiation and 25 AF termination, while the majority of the articles examine the AF perpetuation mechanisms. Finally, 37 articles investigate atrial activation under SR (Weber et al., 2009a, 2009b; Krueger et al., 2013c, 2013d; Voigt et al., 2013).

Apart from electrical remodeling, structural remodeling has also been found to influence the susceptibility to AF. It can be the result of structural heart disease or other conditions such as hypertension, while AF itself can also modify the atrial substrate. As AF progresses, apart from the electrical remodeling, structural remodeling is also present (Wijffels et al., 1995) and interestingly larger atrial size favors reentrant circuits, possibly due to more area available for rotor formation (Zou et al., 2005). The structural remodeling is modeled by considering fibrosis (including collagenous septa, remodeled gap junctions and proliferation of myofibroblast) (McDowell et al., 2013), scars as a result of previous ablation procedures (Gonzales et al., 2014), endo/epicardium dissociation (Gharaviri et al., 2017) and absence of t-tubules (Li et al., 2012). The role of structural remodeling in AF initiation and perpetuation has been thoroughly examined. Zhao et al. (2013b) confirmed that structural remodeling facilitates reentries and multiple wavelets, increasing AF susceptibility. Zahid et al. (2016) described the effect of atrial fibrosis on the appearance of reentrant drivers in patients with persistent AF, and by the use of patient-specific models of the atria, demonstrated that the reentrant activity in fibrotic zones perpetuates AF. The role of the fibroblasts, which can serve both as triggers and substrate to cardiac arrhythmias is described by Koivumaki et al. (2014).

Furthermore, Aguilar et al. (2014) investigated fibroblasts involvement in the maintenance of AF, in HF patients, where they are usually activated. Based on electrophysiological studies in dogs, it was found that a novel fibroblast K^+^ current must be taken into account in the mathematical models to describe the effects of such type of structural remodeling. Finally, Jacquemet and Henriquez (2009) described the impact of microfibrosis progression on electrograms’ fractionation as structural remodeling increases. However, fibrosis distribution can remarkably alter the activation pathways, so there is a great need for accurate atrial substrate mapping using advanced mapping techniques, such as Late Gadolinium (Zahid et al., 2016) and contrast-enhanced mapping (Zhao et al., 2017).

#### Integrating With Structure, Geometry, and Anatomy

##### Detailed anatomical structures

Atrial tissue presents a complex morphology both in terms of structural and electrical heterogeneities. Fiber orientation constitutes a key aspect of atrial anatomy while electrical heterogeneities (such as the fast conduction systems of Bachman bundle, Pectinate muscles, and Crista Terminalis) alter active potential propagation and the way this can influence the initiation and maintenance of AF (Jacquemet and Henriquez, 2009). Vigmond et al. (2001) presented a simple geometrical representation of atrial anatomy toward assessing of the role of the various anatomical structures on the initiation of reentries. In that study, all major anatomical structures of the atrium were considered, including fiber orientation, muscle structures such as crista terminalis and pectinate muscles as well as the orifices of the veins and valves. The outcome model did not constitute an exact representation of the atria, although it reflected the interrelations between the anatomical structures of the atria.

In most studies, fiber orientation was linear whereas, in another study, a sigmoid representation of fiber orientation was adopted (Ashihara et al., 2002). However, in 3D atrial models, fiber orientation is extracted based on imaging data and *ex vivo* tissue analysis (Zhao et al., 2012). Based on successive images from sheep atria, the myofiber orientation was determined by the purpose-development structure tensor analysis whereas the role of the fiber architecture on AF initiation has been studied (Zhao et al., 2013a). Kharche et al. (2014a) constructed a detailed model of rabbit atria from micro-CT images and found that fiber orientation anisotropy serves as a regulator of atrial activation while any modification of myofiber architecture may be pro-arrhythmic.

To develop realistic anatomical models of the atria, Zhao et al. (2008) suggested a methodology, based on *ex vivo* samples from pigs. During the following years, several models were designed to describe a realistic human or animal atrial anatomy (Deng and Xia, 2010; Aslanidi et al., 2011b; Tobón et al., 2013; Li et al., 2014; Ferrer et al., 2015; Zhao et al., 2017), whereas Zhao et al. (2017) proposed a novel 3D computational and structural model of human atria. This model is the output of a systematic analysis. It includes wall thickness, fiber orientation, and transmural fibrosis, and it is the most complete model of the human atrial structure.

Gharaviri et al. (2012a) introduced the novel approach of a dual-layer model for the study of AF. This model has been identified in 11 articles in the current review. Its basic hypothesis is that structural remodeling does not only lead to dissociation within the epicardial layer but also dissociation between the epicardial and endocardial layer promoting AF. AF induces this type of structural remodeling, based on experimental findings from goats (Eckstein et al., 2011), while it was revealed that this dissociation increases AF stability (Gharaviri et al., 2012b, 2017; Verheule et al., 2013). A methodology for the creation of a bilayer model of human atria, using CT images, is proposed by Vigmond et al. (2013), where the difference between RA and LA thickness, as well as the main anatomical structures and fiber orientation, is taken into account. The dual layer approach has also been adopted to examine the effectiveness of different radiofrequency ablation strategies (Bayer et al., 2016).

##### Simulation levels

Electrical activation of each cell results in modification of the electrophysiological properties spanning from the cell to the tissue, organ and body level. According to the scope of each study different observational outcomes were considered, such as AP, activation map, electrogram or ECG. In the cell level simulations, a single cell is considered. In the tissue level studies, the atrial excitation is simulated in a 2D level including several anatomical structures (Seemann et al., 2005; Carrillo et al., 2008). In the organ level studies, 3D models of the atria are considered. 3D structures, like icosahedron (Ellis et al., 2000), or simple atrial representations (Vigmond et al., 2001) have been proposed, while realistic geometrical models of human atria were constructed using MRI (Virag et al., 2002; Aslanidi et al., 2011b; Colman et al., 2013) or CT images (Burdumy et al., 2012). Furthermore, advanced imaging techniques, like late gadolinium-enhanced magnetic resonance imaging (LGE-MRI) allow the construction of patient-specific atrial substrate with fibrosis (McDowell et al., 2012). In body level articles, a realistic model of a human torso is adopted to simulate body surface potentials and the produced ECG. One of the first works in this field is the one of Jacquemet et al. (2006) where an ECG signal representing atrial activation is created, using a geometrical model atrial and torso model, based on MR images. Finally, Krueger et al. (2013a) proposed torso models to simulate surface ECG recordings.

Studying the electrophysiological changes on AP and ion kinetics (Jones et al., 2012; Sanchez et al., 2012; Voigt et al., 2013, 2014) is the main subject of cell level simulations. 2D level studies target at identifying AF drivers, such as rotors and spiral waves (Becerra et al., 2014; Ganesan et al., 2016; Salmin et al., 2016; Duarte-Salazar et al., 2017; Aronis and Ashikaga, 2018) or the effect of spatial resolution on AF drivers detection (Rappel and Narayan, 2013; Roney et al., 2017). The use of patient-specific 3D models can give more insight into the mechanism of AF (Zahid et al., 2016). Those simulations are used for the analysis of the mechanisms for AF initiation (Virag et al., 2002; Chang and Trayanova, 2016; Alcaine et al., 2017), AF stability (Rottmann et al., 2015) or the rotor drifting toward PVs (Calvo et al., 2014) and their anchoring in obstacles, such as fibrotic regions (Morgan et al., 2016; Nguyen et al., 2016; Deng et al., 2017). The detection of rotors is also studied using 3D simulations of human atria (Ugarte et al., 2014; Berenfeld, 2016; Martinez et al., 2016) to investigate the effectiveness of an ablation procedure (Dang et al., 2005; Hwang et al., 2014).

Apart from activation patterns analysis, signals can also be simulated (such as electrograms and ECG recordings). In the 2D or 3D case, the simulated electrograms can serve as a tool for AF drivers identification (Benson et al., 2014; Hummel et al., 2017; Sahli Costabal et al., 2018), atrial activation analysis and complex fractionated atrial electrogram (CFAE) regions detection (Jacquemet et al., 2003; Jacquemet and Henriquez, 2009; Ugarte et al., 2013, 2015; Roney et al., 2016). Jacquemet et al. (2005, 2009) constructed synthetic ECG signals to evaluate ventricular activity cancelation techniques. 3D simulations have been conducted to analyze the ECG characteristics, such as P-wave morphology (van Oosterom and Jacquemet, 2005; Ogawa et al., 2007; Krueger et al., 2013a, 2013c). On the other hand, the inverse problem solutions and body surface recordings were employed for the detection of AF drivers (Pedrón-Torrecilla et al., 2014; Figuera et al., 2016b; Guillem et al., 2016; Pedron-Torrecilla et al., 2016). Finally, an adaptation of the standard ECG systems for the optimization of the atrial signal recording was proposed by Ihara et al. (2007) and van Oosterom et al. (2007).

**FIGURE 7:**
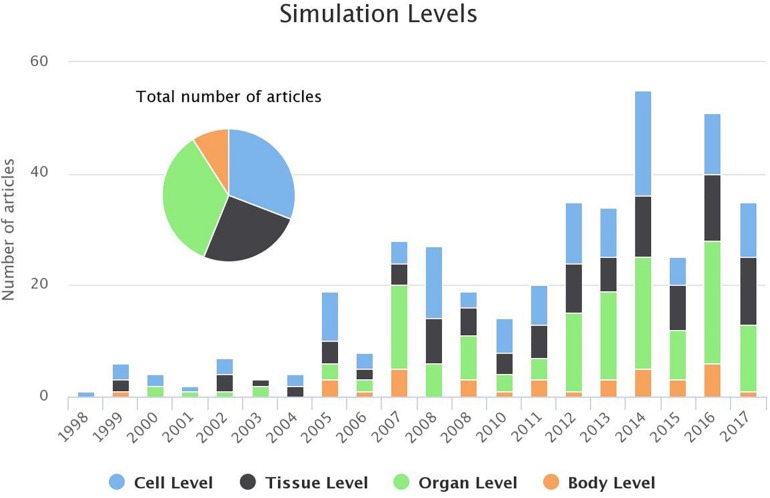
Evolution of the number of studies focusing each of the simulation levels (bar chart) and the distribution of the studies for the last 20 years (pie chart). For 2018 1 study targeted cell level and 2 studies the tissue level (until 1st of March 2018).

As shown in Figure 7, only a small minority of the studies adopt the body level simulations, whereas, more and more studies adopt a 3D realistic model of human atria to study the electrical activation during AF (organ level). Furthermore, the interest in the cell level analysis is persistent, as this constitutes the basis for any simulation protocol.

Electromechanics focus on the estimation of the electrical activation of the atria under the consideration of their mechanical function. Eight articles were found to include the electromechanical function in the simulations. In general, atrial contractility is associated with tissue depolarization as well as with the structural remodeling co-existence. Kuijpers et al. (2007) published the first model that integrates cardiac electromechanics with physiological details. According to this model, stretching of an atrial fiber influences the stretch-activated current ($I_{\text{SAC}}$). The model complies with experimental observations of conduction slowing and blocking as a result of an acutely dilated atria. This model was integrated into a 3D geometrical model of the atria in order to investigate the role of stretch-activated current on the onset and termination of AF episodes (Kuijpers et al., 2009). The role of acutely dilated atria on AF initiation was later reported (Kuijpers et al., 2011). Ni et al. (2014) used an electromechanical model of the ventricles (Adeniran et al., 2013) to study the effect of KCNA5 mutation on the electromechanical function of the human atrial cell while Adeniran et al. (2015) studied the effect of AF-induced electrical remodeling on the electromechanical function of the heart. Finally, a more recent study focused on the analysis of spiral waves when the atrial mechanoelectrical function is considered (Brocklehurst et al., 2017). In this study, one electrophysiological model of human atria (Colman et al., 2013) was modified to include the stretch-activated current, while combined with a mechanical model (Rice et al., 2008) which describes the active force generated in cellular level in response to the electrical signal. This study highlights the effect of mechanoelectrical feedback on AF.

#### Modeling Disease Context and Purpose

##### Rhythm status (SR or AF)

In a healthy heart, the electrical pulse originates from the sinus node and spreads through the atrium (Platonov, 2012), while during an AF episode chaotic activation of atrial myocardium occurs (Yamazaki and Jalife, 2012). In the majority of the papers included in this review, AF was induced and the analysis focused on the identification of AF drivers (Ashihara et al., 2004; Martínez et al., 2014b; Tobón et al., 2014b; Ugarte et al., 2014, 2015; Felix et al., 2015; Martinez et al., 2016; Salmin et al., 2016; Roney et al., 2017; Duarte-Salazar et al., 2017) or on the study of the electrical activation during AF in general. However, analysis of the electrical activation of the remodeled atria during SR was the subject of 52 studies, as this can also improve our understanding of the pathology.

The main property of Sinoatrial Node (SAN) models is their auto-oscillatory behavior (Fink et al., 2011). In most cases, they are used under normal conditions instead of a remodeled substrate. The effect of SAN dysfunction on AF initiation is addressed in several studies. Kharche et al. (2017) developed a 3D electro-anatomical model of the SAN, based on the Fenton-Karma model. The model was based on histological studies, and the role of the SAN exit pathways was examined in terms of arrhythmias initiation. Glynn et al. (2014) adapted a detailed murine SAN model to confirm the restitution hypothesis, which states that the APD is associated with the duration of the previous diastolic intervals. They concluded that cycle length restitution analysis can enhance our understanding on SAN dysfunction and the initiation of arrhythmias. The simulations performed denote that the restitution curve can be useful for the analysis of cell dynamics and dysfunction in SAN. Lin et al. (2017) used a simplified spherical model of LA to investigate the mechanism of SR to AF transition. It was found that during SR, in healthy subjects, SAN to AV node conduction occurs through the interatrial septum via the fast pathway, while during AF, SAN long refractory period protects it from overdrive and the AV node filters the APs which would reach the ventricles (Li et al., 2014).

A common reason of SR usage is to test the reproducibility of the computational models, in both AF and normal SR (Goodman et al., 2005; van Oosterom and Jacquemet, 2005; Cherry and Evans, 2008; Grandi et al., 2011; Burdumy et al., 2012; Krueger et al., 2013a, 2013d; Colman et al., 2016) or to examine differences in activation patterns with or without electrical remodeling (Zhao et al., 2012; Voigt et al., 2014). Simulation under SR, using patient-specific models, including fibrosis, can accurately reproduce local activation (Krueger et al., 2014). The reconstruction of the ECG signals by solving the forward problem of electrophysiology, to understand the genesis of the P-waves, was also found to be very accurate when the patient is in SR (Ferrer et al., 2015; Jacquemet, 2015; Figuera et al., 2016b). Finally, Zhao et al. (2012, 2013a) highlighted that the structurally remodeled substrate and the anatomical heterogeneities can increase the vulnerability to rhythm disturbances.

In addition, considering patients during SR, the effect of antiarrhythmic drugs, such as potassium or sodium channel blockers, on the APD or ionic current characteristics, has been studied (Wettwer et al., 2004; Ehrlich et al., 2008; Law et al., 2010; Aslanidi et al., 2012a; Marshall et al., 2012; Voigt et al., 2013; Ni et al., 2016; Skibsbye et al., 2016; Ellinwood et al., 2017b; Tobón et al., 2017). Ablation success, applied during SR, has been presented (Tobón et al., 2015; Bayer et al., 2016) and the changes occurring in the P-waves after the PV isolation procedure have also been examined (Saha et al., 2016).

##### AF initiation

The conditions, under which AF can be induced, were extensively studied and several approaches have been proposed. In general, a triggering factor, in the presence of an arrhythmogenic substrate, can favor the initiation of PAF. PVs have been reported to present pacemaking automaticity and triggering activity, and thus their role on arrhythmogenicity has been examined (Reumann et al., 2006; Cherry et al., 2007), whereas mathematical models have also been implemented to describe PVs in both animals (Lo et al., 2006; Seol et al., 2008) and humans. Jones et al. (2012) modeled the PVs under the experimental evidence that two types of cells exist in PVs, pacemaking, and non-pacemaking.

Abramovich-Sivan and Akselrod (1999) initiated a new approach, where a model of the atrial cells, able to describe the transition from normal rhythm to AF, is proposed. According to this study, the atrial cells are considered as “pacemaker-like” cells with different cycle length and different type of intercellular connection, where, the effect of the junctions between the different types of cell are taken into account to investigate how the initiation of atrial rhythm disturbances occurs and maintains.

The role of the coupling between anatomical heterogeneous regions, such as CT (Ellis et al., 2000; Reumann et al., 2007; Dorn et al., 2012; Butters et al., 2013; Zhao et al., 2013a), or between the PV and the LA (Aslanidi et al., 2012b, 2013) on the initiation of AF is discussed, whereas atrial contraction and electromechanics are proposed as a mechanism of arrhythmogenesis (Kuijpers et al., 2009). Fibrosis (McDowell et al., 2012; Zhao et al., 2013b; O’Connell et al., 2015) or other anatomical obstacles (Ciaccio et al., 2017), as well as the imperfect ablation lines (Dang et al., 2005) have also been proposed to facilitate AF initiation.

The effect of specific ion currents and electrolyte concentration on arrhythmogeneity was studied (Kuijpers et al., 2006; Aslanidi et al., 2008; Law et al., 2009; Varela et al., 2014; Voigt et al., 2014; Onal et al., 2017) whereas electrical remodeling, induced by AF, and its role on episodes induction is discussed by Colman et al. (2013) and Lee et al. (2016). Furthermore, Morgan et al. (2014) reported that co-existence of structural and electrical remodeling can promote AF initiation.

AF initiation by specific diseases, such as ankyrin-B syndrome (Wolf et al., 2013) and end-stage renal disease, was also examined. In particular, HD effect on AF is highlighted in several studies (Severi et al., 2008, 2010; Krueger et al., 2011) suggesting that HD sessions may be a triggering factor for the onset of the arrhythmia (Vincenti et al., 2014).

Atrial ectopies and heart rate (HR) variations have reported to induce AF. Virag et al. (2002) used three different stimulation protocols. The first one is the S1-S2 protocol, where the SA node stimulation is followed by an ectopic beat located in the RA. The second one is the S1-S2-S3 protocol where an additional ectopic beat is applied in the same areas as the previous one, while the third protocol is a burst pacing protocol of 20 Hz applied in the SA node for several seconds. These protocols, applied in both normal and electrically remodeled substrate, simulate both self-terminated AF episodes as well as more sustained types of AF. It is worth mentioning that most of the studies, analyzing AF drivers, use the S1–S2 protocol for AF initiation (Ramirez et al., 2000; Liu et al., 2007; Tobón et al., 2014b; Ugarte et al., 2015). Shusterman et al. (2003) mentioned that abrupt short-term variations in cycle length, such as atrial ectopic activity and HR variations, lead to spatial irregularities of the wavefront propagation and thus initiate reentries in a simple two-dimensional tissue model. The role of alternans as a proarrhythmic state has also been discussed (Chang et al., 2014; Chang and Trayanova, 2016). Gong et al. (2007) proposed that ectopies originating from the PVs facilitate reentry induction by prolonging the vulnerability window. Hwang et al. (2016) and Hwang et al. (2017) examined the effect of the ANS on arrhythmogenesis where the role of ganglia on the initiation of AF is highlighted. The concept of early warning that can forecast a change of system’s stability is applied in AF electrophysiology (Garcia-Gudino et al., 2017). In this study, a cellular automaton is used to describe atrial conduction on the tissue. Applying different pacing approaches, along with real RR interval data derived from Fantasia database (Iyengar et al., 1996), it was shown that even when complex pacing is introduced, this approach can serve as a predictor of transition from normal rhythm to AF.

Finally, genetic background and gene mutations represent an additional field of research on AF initiation (Carrillo et al., 2008; Seemann et al., 2009; Kharche et al., 2012a; Whittaker et al., 2017).

##### Modeling the interventions for AF termination

Atrial fibrillation termination approaches include pharmacological treatment, ablation procedure, and electrical cardioversion. The first study identified on defibrillation usage for AF termination is the one by Boegli et al. (2000) where different defibrillation techniques were tested for their effectiveness. Kharche et al. (2012b), using a 3D realistic model of human atria, observed that low energy cardioversion efficacy depends on atrial anatomy. It has also been mentioned that in patients with no electrical remodeling lower cardioversion energy and shorter duration must be applied (Kharche et al., 2013). Rusu et al. (2014) proposed that pacing therapy must be patient-specific, due to its influence by the presence of heterogeneities in vagal activation and repolarization. On the other hand, it has been suggested patient-specific management of defibrillation (Kharche et al., 2015; Luca et al., 2016).

Dang et al. (2005) evaluated the outcome of the radiofrequency ablation and studied the effect of imperfect lines. In this study, the significance of the biophysical models’ application on the development of new ablation approaches is highlighted. The simulation results were confirmed with clinical data. The identification of the optimal ablation line is the subject of several studies which point out the need for tailored ablation lines for each patient (Rotter et al., 2007; Ruchat et al., 2007a, 2007b, 2007c; Reumann et al., 2008; Kwon et al., 2013; Hwang et al., 2014; Zhu et al., 2014; Rappel et al., 2015; Bayer et al., 2016). During the ablation procedure, in most cases, the PVs are isolated. However, fibrotic region ablation is also proposed (Ashihara et al., 2012) whereas other studies suggest other approaches which can guide the ablation procedure (Benson et al., 2014; Tobón et al., 2014b; Carrick et al., 2016; Kuklik et al., 2016; Li et al., 2016). In a 3D model of LA, the ablation of ganglia seems to terminate AF (Hwang et al., 2017). Interestingly, the effect of the poor contact of the electrodes with the tissue on AF drivers identification is discussed, and a methodology for signals reconstruction is proposed by Green et al. (2017).

Apart from the approaches mentioned above, drug treatment is also followed for AF termination. Most of the antiarrhythmic drugs, acting on atrial myocardium, may also have an effect on ventricular ion channels. State-dependent $N\,a^{+}$-channel blockers (class I antiarrhythmic drugs) maximize their effect on the atrium while at the same time the influence on the ventricles is minimal. AF-selectivity of such drugs is the topic of Aguilar-Shardonofsky et al. (2012). According to this study, increased ventricular proarrhythmia correlates with drug effectiveness close to 100%, while in case of minimum ventricular proarrhythmia, AF termination rates were low. Furthermore, several potassium channel blockers were also analyzed, in terms of computer models, in order to examine their effect on AF termination (Scholz et al., 2013; Tobón et al., 2014a; Aguilar et al., 2017; Ellinwood et al., 2017a). Ni et al. (2017b) compared the effects on atrial and ventricular activation of these two types of blockers combination to single channel blockers usage. The human heart simulation results showed that synergetic antiarrhythmic treatment leads to more effective termination of reentries, compared to single channels block, while the side effects in the ventricles are limited. Moreover, analyzing inter-subject variability on AP, distinguishes drug responders from non-responders (Liberos et al., 2016).

The self-terminating nature of the arrhythmia has also been studied (Uldry et al., 2012), however, its transient nature is the main barrier while an analysis framework has been proposed (Lin et al., 2017). It was found that in low complexity AF, the spontaneous termination process lasted 3.2 s and was initially started in the LA, while the last fibrillatory signal was detected in the RA. On the other hand, in complex AF, the termination process is less predictable. Additionally, the role of electro-mechanical feedback on AF self-termination is discussed by Kuijpers et al. (2009).

#### Evolution of Models

Computational modeling evolution can be studied by network theory. The components, involved in AF modeling, are considered as nodes of a network, whereas, the connections between them represent the studies they are reported in. Two distinct periods (1998–2007 and 2007–2018) were considered, and two metrics, describing the network complexity were extracted. The first metric is the average degree (*AD*) of the graph, which is defined as the average connections between the nodes, while the second one is the network density, defined as $D=\frac{2\,E}{N\,\left(N-1\right)}$, where *E* is the number of edges and *N* the number of nodes.

According to the analysis *AD* was 16.96 during the first decade its value increased to 21.52 during the second decade, demonstrating an increase in network complexity as more factors were involved in the computational modeling. *D* was also increased during the second decade, from 0.707 to 0.897. These results suggest that the simulation experiments are getting more and more complex, taking into account additional factors which influence AF.

**FIGURE 8:**
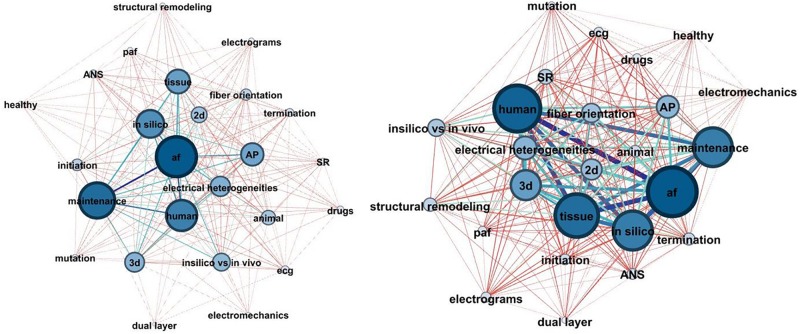
The network chart depicted the evolution of AF modeling complexity during the last 20 years (1998–2008: left, 2009–2018: right). The nodes of the network represent the basic components of the AF modeling concept, and the edges represent their connections. The stronger the prevalence of the nodes and edges, the higher their thickness, and the darker their color.

As seen in Figure 8, the study of subjects such as the dual layer, the electromechanics and the involvement of ANS, gained the attention of the research community during the second decade. Additionally, 3D model simulations also doubled their frequency which can be attributed to the improvement of imaging techniques and the creation of detailed anatomical models of the atria. Finally, it is observed that more studies focus on atrial activation analysis during SR and not only while on AF.

On the other hand, the proportion of studies, conducting an AP model analysis seems to be the same between the two decades, implying a stable trend in the analysis of APs.

### AF Models: What Do We Learn About ECG and P-Wave Morphology

One of the great challenges in the field of computational modeling is to translate the output of the simulation studies into clinical practice (Trayanova and Chang, 2016). ECG recordings constitute an accurate non-invasive tool for heart activity assessment which is extensively useful in daily practice. ECG signals extraction from computational studies and vice versa would improve AF pathophysiology understanding and thus optimize clinical decision and treatment strategy. In the present review, several approaches for ECG signal reconstruction have been identified.

An attempt to extract a pseudoelectrocardiogram (pECG) was made by Namba et al. (1999) where a point in a realistic distance of the excitable tissue was considered and the pECG was calculated as the integration of all the membrane potentials, assuming a homogeneous and zero resistance area. In this work, it was found that the spiral wave results on a low Atrial Fibrillation Interval (FF) while under sodium channel blockers the FF intervals increase. Aslanidi et al. (2011b) described the development of the 3D virtual human atria which is a multi-scale detailed computational model. The anatomical model of the atria was based on MR images where the main anatomical structures, with different electrical characteristics, and fiber orientation were included. The 3D virtual atria model was adjusted in a torso model, enabling several activation patterns simulation and ECG extraction. A detailed anatomical model of human atria and torso, based on histological data and MR images, is also described by Ferrer et al. (2015). The concept of this work was to create a multi-scale framework used to explain, from an electrophysiological point of view, findings that are detectable in P-waves and body surface potential maps. P-wave morphology and duration were observed to be highly influenced by the conductivity properties of the torso and the fiber orientation in the atria.

Kuijpers et al. (2011) tested the effect of mechanical function on AF initiation and its effect on the extracted ECG was estimated. In another work from Krueger et al. (2013d) the personalized anatomical model in terms of the location of the interatrial bridges is studied. MRI images were used to create a personalized anatomical model while local activation time (LAT) maps were recorded using electroanatomical mapping systems [Ensite NavX (St. Jude Medical, St. Paul, MN, United States) or Carto (Biosense Webster, Haifa, Israel)] before the ablation procedure. Those LAT maps were used to localize the interatrial bridges in each patient. The simulation results, during SR, denote personalized interatrial bridges consideration can improve simulated and real body surface potentials concordance.

In an ECG signal atrial activity, especially atrial repolarization is hidden by the much higher amplitude of the ventricular electrical activity. In this respect, the cancelation of the ventricular wave is of great importance. Validation of ventricular activity cancelation algorithms is feasible employing computational modeling and ECG signal reconstruction. Jacquemet et al. (2009) created a realistic 3D model of human atria and torso, based on MR images, and the signal, reflecting atrial activity, was reconstructed in the points that a standard ECG lead is measured. That signal has later been summed with QRST complexes recorded during SR, adapting the RR intervals accordingly. This work does not focus on the analysis of the F-waves, but it provides the methodological framework for the evaluation of QRST cancelation techniques.

The standard ECG-lead systems, such as the 12-lead ECG, were designed in such a way to capture the ventricular activity effectively. Thus, these lead systems may be sub-optimal in terms of atrial activity detection. A few studies have been conducted (Ihara et al., 2005, 2007) proposing the modification of these systems toward the effective capturing of atrial activity. Additionally, van Oosterom et al. (2007) suggested a modification of electrodes’ placing vectorcardiographic (VCG) signals acquisition in AF patients, as, according to the authors, the Frank lead system seems suboptimal for atrial activity monitoring during AF. These studies are based on 3D models of atria and torso, where the ECG on the body surface is calculated using simulations.

Electrocardiography and vectorcardiographic recordings have been analyzed to identify AF drivers as well as to characterize atrial activation complexity. An interesting visual representation of the atrial activation, using the time course of the VCG dipole components, is based on a sphere where the main vessel and vein orifices were included. The trajectory of the atrial activation demonstrates the differences between SR and different types of AF (Jacquemet et al., 2006) while the extracted spatial characteristics can localize stable AF drivers in the atria (Lemay et al., 2007b, 2009). Lemay et al. (2007a) proposed that the existence of multiple AF drivers in the atrium can be revealed through the analysis of the full spectrum of the ECG signal instead of the analysis of the dominant frequency only. Duchêne et al. (2009) used a multiple frequency tracking algorithm to examine whether ECG signals analysis can provide information related to AF drivers complexity. Furthermore, rotor activity detection using spherical atrial and torso model and surface phase maps extraction is proposed in Rodrigo et al. (2014a, b). Finally, Ni et al. (2017b) examined the effect of antiarrhythmic drugs on the power spectrum of the simulated ECG.

In the surface ECG, atrial depolarization is mainly represented by P-wave. While AF is characterized by the absence of P-waves in the ECG signals, during SR, this complex can provide information related to the underline substrate. It is known that the first part reflects the activation of the RA and the second part the activation of the LA (Michelucci et al., 2002). According to Platonov (2012), the origin of the sinus rhythm, the shape and the size of the atria can affect the morphological characteristics of the P-waves. Solving of the forward or the inverse problem of the electrophysiology helps to translate the P-wave characteristics on tissue level phenomena. However, it is worth mentioning that both the inverse and forward approaches require a detailed representation of the torso as this affects the precision of the results (Bear et al., 2015).

#### Forward Problem – P-Wave Estimation

The estimation of the body surface potentials based on the epicardial ones, define the forward problem of electrophysiology. The key work from van Oosterom and Jacquemet (2005) used the Equivalent Double Layer (EDL) source model to simulate the atrial activity of the human body. The proposed approach was applied both during SR and AF. During SR, the application of the EDL model presented high correlation with the actual recorded signal, primarily related to the P-wave morphology. Furthermore, during AF, simulated signals were also highly correlated with the signals that are typically recorded in AF patients.

Krueger et al. (2013c) presented a framework for the generation and validation of multiscale cardiac modeling. This study describes how *a priori* knowledge, such as fiber orientation and electrophysiological models, can be enhanced by information derived from ECG characteristics like P-wave duration or HR, electrolytes concentrations and information from cardiac and thoracic MRI. The availability of LATs and ECG recordings can be used for models evaluation. As highlighted, HR must be considered during the simulation process as it has a significant impact on atrial repolarization. Additionally, P-wave morphology was properly reproduced only in specific leads, while P-wave duration is accurately computed in any lead. Krueger et al. (2013a) simulated P-wave as well as T-wave, based on the patient-specific detailed anatomical model. The simulated P-waves and T-waves morphology fitted well with the experimental recordings in both physiological and AF-remodeled conditions.

A typical example of macroscopic phenomena translation is to predict AF recurrences by analyzing the P-wave shortening observed in patients undergoing PVs isolation. P-wave duration reflects the interatrial conduction time while in AF patients the heterogeneous atrial propagation results in prolonged P-waves. Ogawa et al. (2007) used signal-averaged P-wave duration, P-wave dispersion and root-mean-square voltage of the terminal 20 ms (RMS20) of the averaged P-wave, to predict AF recurrences after successful PV ablation. Comparing real data from patient underwent cardiac ablation with P-wave generated by mathematical simulation of realistic human atria, it was found that the elimination of PV sleeve results on shortening of the P-wave duration and modification of its morphology. Jacquemet (2015) discussed LA and RA substrate contribution on P-wave morphology confirming that P-wave reflects any structural modification of the atrial substrate.

An effort to interpret the atrial substrate mechanisms, which lead to changes in P-wave duration after the ablation procedure, was performed by Saha et al. (2016). Using mathematical models of human atria and torso, VCG and 16-lead ECG were reconstructed before and after PVs isolation. According to the results, P-wave shortening is detected in leads V3 and V4 whereas these changes were more apparent when larger PV areas were isolated. Furthermore, the P-wave area was increased in V1, while decreased in V6. Additionally, possible reconnections in the PVs could be identified, and thus the 16-lead ECG can be used as a tool for PVs reconnections detection in patients undergone ablation.

P-wave terminal force in lead V1 (PTF-V1) has been proposed as an AF risk marker. PTF-V1 is the product of the duration and the amplitude of the terminal negative part of the P-wave (Morris et al., 1964). Loewe et al. (2016) studied the effect of the Early Activation Sites (EAS), as well as, their proximity to inter-atrial connections (IACs) on the PTF-V1. The term EAS is defined as the area where the sinus node excitation is captured by the myocardium. An inter-personal variability, as well as, a variation over time on the same individual has been observed. Based on experimental findings from dogs, this variation over time is suggested to reflect the vagal activation. On the other hand, the location and the conductive properties of the IACs are known to vary among subjects. Several anatomical models and multiple EAS consideration have shown that these two factors affect PTF-V1.

Alday et al. (2014, 2015) examined the effect of ectopic foci on P-wave morphology. In the latter study, the P-wave morphology is examined in terms of polarity (positive, negative, and biphasic). Under the assumption that a negative deflection on an ECG depicts a wavefront traveling away from the location where the electrodes are placed, the torso and the atria were separated into four quadrants. The analysis of the P-wave polarity in each of the quadrant can reveal the area from which the ectopic foci originates, while the implemented algorithm demonstrated high success rates on the localization of the ectopic source.

As mentioned, alternations on the ionic currents during HD sessions can induce PAF episodes. Observational studies have shown P-waves prolongation during HD sessions implying intra-atrial conduction slowing. A computer model describing the ionic concentrations of a patient during HD has been used to explain this macroscopic phenomenon (Severi et al., 2010). $K^{+}$alternations (hypokalemia) result in conduction velocity reduction and ERP shortening. Krueger et al. (2011) confirmed these findings, where simulated ECGs were extracted, using realistic models of human atria and torso.

Finally, Zhu et al. (2014) examined P-wave morphology to check the effectiveness of 4 ablation protocols, on cAF treatment. Without providing any details on the evaluation of P-wave morphology, it is mentioned that simulated P-waves were similar after the application of different ablation protocols. The authors concluded that the Maze III approach is effective, and the transmural ablation has the same effectiveness as the non-transmural one. Table 3 summarizes the features mentioned above for the analysis of the P-wave using computational modeling approaches.

**TABLE 3:** Features used toward the analysis of the P-wave, during SR, using computational modeling approach.

**Article**	**Intervention**	**Feature**	**Goal**
Ogawa et al.,2007	ablation	PWD RMS20 P-wave dispersion	Prediction of AF recurrences
Krueger et al.,2011	HD	P-wave duration	HD-induced changes
Krueger et al.,2013a	–	PWD Ta-wave duration Ta-wave amplitude	Study atrial repolarization
Krueger et al.,2013c	–	PWD	Framework for personalized models
Alday et al.,2014	–	P-wave morphology (polarity)	Localize ectopic foci
Alday et al.,2015	–	P-wave morphology (polarity)	Localize ectopic foci
Ferrer et al.,2015	–	P-wave morphology duration amplitude	Multi-scale framework
Saha et al.,2016	ablation	PWD P-wave area duration of the positive part of P-wave	Changes after ablation on P-wave
Loewe et al.,2016	–	P-wave terminal force in lead V1 (PTF-V1)	Inter and intra-individual variation of the P-wave morphology

#### Inverse Problem

Estimation of bioelectric source potentials using remote electric field measurements is known as the inverse problem of electrophysiology, and it has been applied in both SR and AF scenarios (Pedrón-Torrecilla et al., 2014). The accuracy of the extracted results is a crucial topic and several studies focused on the implementation of algorithms able to reconstruct activation patterns matching with the observed ones (Pedron-Torrecilla et al., 2016). Zemzemi et al. (2012) proposed one such approach based in machine learning techniques where the simulated ECGs, computed by the consideration of atria and torso model, were used for the training of the algorithms while the results were examined in terms of reproducibility. One of the application areas of this field is the identification of AF drivers in the atria (Guillem et al., 2016). On the other hand, different methods of estimating epicardial potentials, dominant frequency, phase maps, and singularity points locations, were examined for their accuracy (Figuera et al., 2016a, 2016b).

## Discussion

Computational modeling is an emerging area of research useful to understand the mechanisms involved with pathophysiology, diagnosis, and treatment of human diseases (Bers and Grandi, 2011). This article presents the results of systematic scoping review related to multi-scale computational modeling of AF. In the context of this review, two databases were searched and 301 articles, published during the last 20 years, were identified as relevant for our purposes.

Atrial fibrillation is a complex arrhythmia, and the electrophysiological mechanisms for its initiation and maintenance are not clearly understood. In general, a triggering factor, as well as an arrhythmogenic substrate, must coexist for AF initiation. The complicated nature of AF is reflected in the significant number of studies included in the current review and the numerous factors to be considered on simulation protocols designing. The advances in imaging technologies led to the creation of detailed anatomical models, depicting the structural and electrical heterogeneities of the atria whereas, several mechanisms involved in arrhythmogenesis have been modeled related to electrical and structural remodeling. The computational approaches move toward more detailed models, incorporating multiple factors, such ANS effect, atrial electromechanical, the endo and epicardium layer dissociation, fibrosis and the genetic substrate. This progress will lead to patient-specific models creation (Krueger et al., 2013b) which can be useful in real-life cases analysis.

The observation of the number of articles published over time reveals an increased interest in the field of computational modeling of AF while this interest is projected to continue. Furthermore, the number of papers focusing on *in silico* AF models is increasing, and multiple scales are being analyzed, ranging from cell to tissue and organ level. In time to come, the advances of mapping and imaging techniques as well as high-performance computing engineering, will force computational models to adopt new elements to improve the repeatability and consistency with clinical observations (Trayanova and Chang, 2016). The main challenge is to transform the experimental knowledge into clinical practice in order to serve as a tool for physicians to perform for clinical interventions (Trayanova and McDowell, 2018). The importance of Physiome project (Hunter and Borg, 2003) and the Virtual Physiological Human (VPH) initiative (Viceconti and Hunter, 2016) and the positive effect they had on the increased interest on computational modeling must be highlighted.

Apart from atrial substrate analysis during AF, some articles focus on atrial excitation during SR. Computer simulations can help interpret the findings from ECG and P-wave analysis. As Dössel et al. (2017) mentioned, merging of biosignal analysis with computer modeling can enhance our understanding of AF. Atrial geometry (Ihara et al., 2007) as well as the modification of anatomical IACs have also shown the ability to change the morphology of the P-waves (Loewe et al., 2016) while the effect of structural remodeling on the P-wave (Jacquemet, 2015) such as its shortening after the PV isolation procedure (Saha et al., 2016), has also been reported. Furthermore, P-wave is supposed to be reformed by ectopic foci (Alday et al., 2015), suggesting that any deflection from the SA node excitation can alter the conduction routes. P-wave morphology depends on (1) the origin of the SR as well as (2) the conduction route (Platonov, 2012). However, many studies have revealed the existence of a distinct secondary P-wave morphology in PAF patients (Filos et al., 2017), while the percentage of beats matching the main or the secondary P-wave morphology predict the outcome of PV isolation (Huo et al., 2015) or the chance for AF initiation (Martínez et al., 2012). Monfredi et al. (2010) investigated whether the variability in the P-wave morphology can be attributed either on a transient switch from one group of SAN pacemaker cells to another or on multiple activation routes. Studies in dogs revealed that ANS, and in particular the different branches shifted the EAS in different regions (Platonov, 2012). Apart from the effect of the ANS, additional factors which alter on different time scales, such as electromechanics and subcellular phenomena like metabolism, must also be included in the analysis of the P-wave morphology variability. However, there is a need to consider longer simulations times and analysis of more beats to capture the transient modification of the P-wave morphology.

However, atria must not be regarded as being isolated from the rest of the heart. The effect of AV node on AF, as well as the role of accessory pathways in conditions, such as Wolff–Parkinson–White Syndrome, must also be considered. Recently, the predictive value of corrected QT interval (QTc) prolongation, a metric of ventricular repolarization, on the AF incidence was highlighted (Zhang et al., 2018). However, this metric can be considered as a surrogate marker. Future simulation protocols should also incorporate the role of other anatomical structures of the heart to investigate the distributed conduction in the atrial substrate which is implied by the variable P-wave morphologies.

Any effort made in the field of computational modeling must seriously consider how the outcome can be used in clinical practice (Grandi and Maleckar, 2016). Guided ablation is one of the main topics influenced by the advances in cardiac simulations (Zhao et al., 2015; Jacquemet, 2016). Computer models can identify AF drivers effectively when the effect of multiple factors on AF, such as structural heterogeneities and electrical remodeling, is considered. In the future, the analysis will be based on personalized atrial models since the evaluation of theoretical ablation procedures in such models showed their effectiveness on AF treatment. Upon application of these techniques in real patients, computational modeling is expected to optimize ablation procedures (Boyle et al., 2017). Furthermore, the development of more effective drugs with substantial atrial selectivity is foreseen (Grandi and Maleckar, 2016).

### Limitations

Studies were limited to the AF while other pro-arrhythmic states were not included in the review. Furthermore, since one of the inclusion criteria was that the term “AF” needed to be indexed as a keyword, we recognize that some pioneering works on the field of computational modeling may be excluded. A typical example is the work from Vigmond et al. (2004), which was excluded because it was not indexed with the term “AF” as a keyword neither in PubMed nor in Scopus libraries, despite the fact its focus is on atrial arrhythmogenesis, and AF can be considered as a part of it. However, the fact that all the remaining inclusion criteria are met denotes the effectiveness of the query for the description of the field of computational modeling. Finally, the modeling of the AV node, which serves as a filter for the atrial AP to prevent the arrhythmia to be diffused into the ventricles, was not considered here, and thus its effect on the initiation and maintenance of AF was not studied.

## Conclusion

Computer models become more and more complex as they include an increasing number of parameters. The evolution of the complexity, depicted in Figure 8, clearly reflects this pattern. The consideration of ANS branches, in terms of ganglia modeling, and the development of detailed representations of the anatomical heterogeneities, using MRI techniques, improved the reproducibility of the simulations compared to clinical observations. The advances on genomics is also an aspect which is under consideration. The advances on high-performance computing can overcome the computation load and the barriers introduced and thus it can allow the observation of more macroscopic events, such as the transition of the conduction routes which are reflected in the existence of multiple P-wave morphologies in AF patients during SR. Eventually, electrophysiological models of the heart will be used to optimize AF treatment and improve the quality of life in this group of patients.

## Author Contributions

The study was initiated by the previous works of all the authors. DF and IC defined the inclusion/exclusion rules. DF, IC, and DT initiated the design, development, and synthesis of the studies and received feedback from NM and VV. DF and IC coordinated the writing of all drafts of the manuscript and all other authors contributed to the submitted versions of the manuscript. All authors have read and agreed to the manuscript being submitted as it is.

## Conflict of Interest Statement

The authors declare that the research was conducted in the absence of any commercial or financial relationships that could be construed as a potential conflict of interest.
